# Complete mitochondrial genomes of two snail mite: *Riccardoella tokyoensis* and *R. reaumuri* (Acariformes, Prostigmata, Ereynetidae)

**DOI:** 10.1080/23802359.2021.1915718

**Published:** 2022-02-07

**Authors:** Shimpei F. Hiruta, Tsukasa Waki, Satoshi Shimano

**Affiliations:** a Center for Molecular Biodiversity Research, National Museum of Nature and Science, Tsukuba, Japan; b Faculty of Science, Toho University, Chiba, Japan; c Science Research Center, Hosei University, Tokyo, Japan

**Keywords:** Prostigmata, Ereynetidae, *Riccardoella*, snail mite, complete mitogenome

## Abstract

We determined the mitochondrial genome sequences of two snail mites, *Riccardoella tokyoensis* and *R. reaumuri*. The length of the entire mitogenome of these two species is 15,078 bp and 15,148 bp long, respectively. Both of them contain 13 proteins, two rRNAs, and 22 tRNAs for a total of 37 gene products. The gene order of *Riccardoella* is able to explain by a single rearrangement event from that of other Eupodina species; the whole region, including both rRNA genes and control region (CR), is inverted at the same position. The CR including a tandem repeat region in both of the mitogenomes of *Riccardoella* species.

So far, mites parasitizing land snails are known from three different families (Fain and Barker 2003). The first is the Eupodidae, mostly soil-borne, but only *Eupodes voxencollinus* Thor, 1934 has been found in the pallial cavity of bulimulid and helicid gastropods (Polaco and Mendl 1988) though this parasitism was probably accidental (Fain 2004). The second is the Trombiculidae, where larvae of *Endotrombicula vanmoli* (Vercammen-Grandjean & Benoit, 1971) have been reported to be deeply embedded in the soft integument (Fain 2004). The last family is Ereynetidae, and although unidentified species of the genus *Boydaia* also have been found on snails of which infection was also probably accidental (Polaco and Mendl 1988; Fain 2004), the most famous and globally distributed mite genus is *Riccardoella* Berlese, 1923 (Ereynetidae). This genus currently consists of eight species, and six of them have been recorded from the lungs of terrestrial gastropods (Turk and Phillips 1946; Fain and van Goethem 1986; Fain and Klompen 1990; André et al. 2004; Waki et al. 2018). The remaining two species were collected only from soils and thought to be free-living (Fain and van Goethem 1986; André et al. 2004). Among the mites belonging to the genus, *Riccardoella limacum* (Schrank, 1776) is known to parasitize several snails and sometimes cause a severe problem with edible snail farming (Baur and Baur 2005; Schüpbach and Baur 2008).

The phylogenetic relationships of Eupodides, including these snail mites, have been studied based on morphological information up to the early 2000s (Lindquist 1996; Andre and Fain 2000). Recent higher-level molecular phylogenetic studies indicated the uncertainly of the monophyly of Supercohort Eupodides. Eupodides was a polyphyletic group in Dabert et al. (2016) and one of the superfamily Eriophyoidea was unstable among the markers in Klimov et al. (2018). Waki et al. (2018) used COI partial sequences to clarify the phylogenetic position of the genus *Riccardoella* among Eupodides. The snail mites were situated inside the superfamily Tydeoidea. However, the monophyly of the two superfamilies themselves and the families' relationships consisting of the superfamilies was unclear.

Thus, further molecular markers and more taxon sampling are urgent to elucidate phylogenetic relationships and genetic structure for revising taxonomy and species diversity in Ereynetidae mites. However, there was no mitogenome record for the superfamily Tydeoidae, and only five mitogenome sequences were reported from species of supercohort Eupodides. Hence, we choose two *Riccardoella* species for the representative of the superfamily and determined the whole mitogenome sequences by shotgun sequencing for both species.

Samples of *Riccardoella tokyoensis* were collected with host species (*Tauphaedusa tau*) at Rinshi no Mori Park, Tokyo (35.6243 N 139.7035 E). For *R. reaumuri*, host snails (*Euhadra callizona*) were collected at Nishizato, Shizuoka (35.1137 N 138.4168 E). Total DNA was extracted using DNeasy Blood & Tissue Kit (QIAGEN, Hilden, Germany) and processed by QIAseq FX DNA Library kit (QIAGEN, Hilden, Germany). Paired-end sequencing (300 cycles) was conducted by the National Museum of Nature and Science, Tokyo on MiSeq, with inserts of ca. 50–200 bp, for a total of ca. four million reads. Assembly was performed using CLC Genomics Workbench ver. 12 (QIAGEN, Hilden, Germany) with the default setting. The ambiguous part of the contig was proofread by 3500 xL Genetic Analyzer (Thermo Fisher Scientific Co., Waltham, MA). Gene identification was made using MITOS web server (Bernt et al. 2013) and ARAGORN ver. 1.2.38 (Laslett and Canbäck 2004, 2008). Voucher specimens with extracted DNA were deposited at the National Museum of Nature and Science, Tokyo (NSMT-DNA 50369 and 50371).

The determined mitogenome length of *Riccardoella tokyoensis* Waki & Shimano, 2018 (GenBank/DDBJ/EMBL accession number LC601992) and *R. reaumuri* Fain and van Goethem, 1986 (LC601993) is 15,078 bp and 15,148 bp long, respectively. Both of them contain 13 proteins, two rRNAs, and 22 tRNAs for a total of 37 gene products. The overall A + T content of the *R. tokyoensis* and *R*. *reaumuri* mitochondrial genome is 79.9% and 81.6%, respectively, which is slightly higher than the ordinal range among Eupodina species (66.3–78.6%). In the mitogenome of *R. tokyoensis* and *R. reaumuri*, ATP8 starts with ATC/ATT codon, respectively. The gene order of *Riccardoella* is able to explain by a single rearrangement event from that of other Eupodina species. The whole region, including both rRNA genes and the control region (CR), is inverted at the same position. CR including tandem repeat region in both of the mitogenomes of *Riccardoella* species.

The maximum-likelihood (ML) phylogenetic analysis based on translated amino-acid sequences of 13 protein coding genes was conducted by RAxML-NG ver.1.0.1 (Kozlov et al. 2019) with bootstrap analyses of 1000 replicates. The phylogenetic tree also with posterior probability from Bayesian analyses (BAs) conducted by MrBayes 3.2.6 (Ronquist et al. 2012). In the tree, *R. tokyoensis* and *R. reaumuri* made a sister clade with species belonging to the family Bdellidae with high nodal support value (Figure 1). The species of the family Eriophyidae formed a polyphyletic group, nested with *Rhinotergum shaoguanense* Xue, Song & Hong, 2009 (Diptilomiopidae). Although additional OTUs are needed, this mitogenome would be useful for reconstructing higher systematics of Eupodides mites.

**Figure 1.**
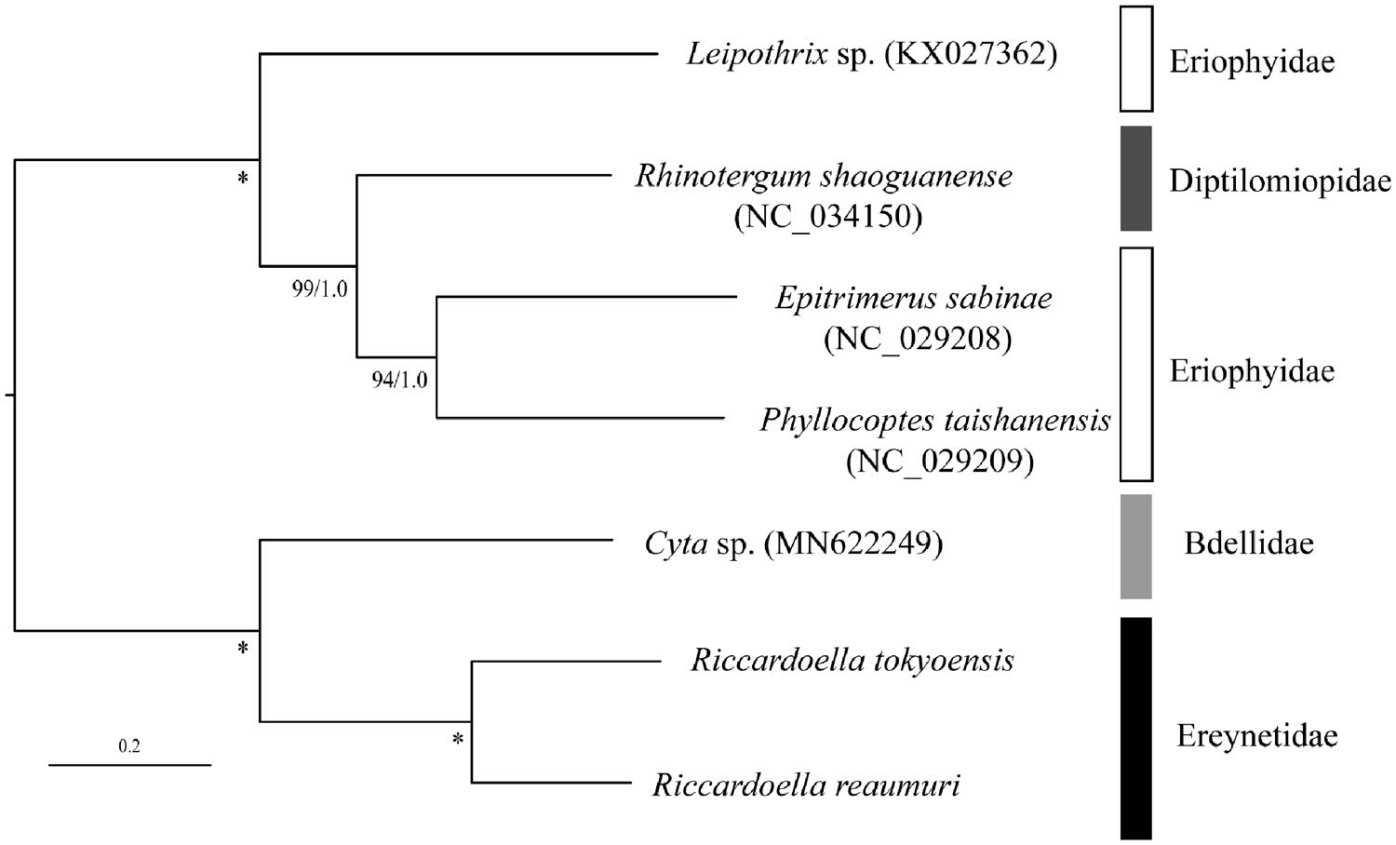
Maximum-likelihood tree based on the concatenated nucleotide sequence of 13 protein-coding genes of *Riccardoella tokyoensis* (LC601992) and *R. reaumuri* (LC601993), five further Eupodides species. Accession numbers of the mitogenome sequences for each taxon used in the phylogenetic analysis are shown in parentheses. Nodal values are ML bootstrap support (BS) values and BA posterior probabilities (PPs). *100% BS and 1.0 PP. The scale bar indicates branch length in substitutions per site. PartitionFinder 2.1.1 (Lanfear et al. 2017) was used to determine the best partitioning scheme and the substitution model with branch lengths linked and a greedy search algorithm (Lanfear et al. 2012). The optimal partitioning strategy and evolutionary models consisted of thirteen genes data set for ML analyses were as follows; partition 1 (ND2 and ND3), partition 2 (CYTB, COII, and COIII), partition 3 (ATP6), partition 4 (ATP8), and partition 5 (ND6) with mtZOA + G+F; partition 6 (COI) with mtART + G+F; partition 7 (ND1, ND4, ND4L, and ND5) with mtZOA + I+G + F. For BA analysis, partition 1 (COI) and partition 2 (CYTB) with mtREV + G; partition 3 (rest of 11 PCGs) with GTR + I+G.

## Data Availability

The data that support the findings of this study are openly available in the National Center for Biotechnology Information database (NCBI/GenBank) at https://www.ncbi.nlm.nih.gov/, accession numbers LC552026 and LC552027. Voucher specimens with extracted DNA were deposited at the Center for Molecular Biodiversity Research, National Museum of Nature and Science, Tokyo (Makoto Manabe; manabe@kahaku.go.jp) under the catalog numbers NSMT-DNA 50369 and 50371.
